# 260. Durability of Antibody Responses to RSV in Older Adults Following Hospitalization for Acute Respiratory Infection

**DOI:** 10.1093/ofid/ofad500.332

**Published:** 2023-11-27

**Authors:** Caroline Ciric, Binh Ha, Ashley Tippett, Laila Hussaini, Luis W Salazar, Olivia Reese, Wensheng Li, Hui-Mien Hsiao, Theda Gibson, Elizabeth Begier, Robin Hubler, Qing Liu, Bradford D Gessner, Benjamin Lopman, Nadine Rouphael, Satoshi Kamidani, Evan J Anderson, Larry Anderson, Christina A Rostad

**Affiliations:** Emory University School of Medicine, Atlanta, Georgia; Emory University, Atlanta, Georgia; Emory University, Atlanta, Georgia; Emory Univeristy, Atlanta, Georgia; Emory University, Atlanta, Georgia; Emory University, Atlanta, Georgia; Emory University School of Medicine, Atlanta, Georgia; Emory University School of Medicine, Atlanta, Georgia; Emory University School of Medicine, Atlanta, Georgia; Pfizer Vaccines, Dublin, Dublin, Ireland; Pfizer Inc., Collegeville, Pennsylvania; Pfizer Inc., Collegeville, Pennsylvania; Pfizer Biopharma Group, Collegeville, Pennsylvania; Rollins School of Public Health | Emory University, Atlanta, Georgia; Emory University School of Medicine, Atlanta, Georgia; Emory University School of Medicine and Children's Healthcare of Atlanta, Atlanta, Georgia; Moderna, Inc., Atlanta, Georgia; Emory University School of Medicine, Atlanta, Georgia; Emory University School of Medicine and Children's Healthcare of Atlanta, Atlanta, Georgia

## Abstract

**Background:**

Respiratory syncytial virus (RSV) is a common viral pathogen identified in older adults with acute respiratory infections (ARIs). Data describing the durability of immune responses to RSV are limited but suggest responses may be short-lived. In this study, we assessed longitudinal antibody responses following RSV-associated hospitalization for 3 years.

**Methods:**

Between September 2018 and March 2020, adults ≥ 50 years of age hospitalized with ARI at two Emory University hospitals who had a positive RSV test via PCR were enrolled. A nasopharyngeal swab, oropharyngeal swab, and 10mL blood were collected at enrollment (V1), 30 days after enrollment (V2), and in the 4 weeks prior to the start of the influenza season(s) for three years after enrollment (1 yr, 2 yr, 3 yr). Syndrome-positive, RSV-negative controls were also enrolled. Serum samples were analyzed for RSV A/B lysate antigen by enzyme-linked immunosorbent assay (ELISA) and end-point titers were interpolated to a standard curve. Geometric mean titers (GMTs) were calculated, and statistical comparisons of log-transformed titers were performed using a mixed effects model in GraphPad Prism v9.0.

**Results:**

Of the 30 enrolled participants who completed follow-up, there were 20 RSV-positive cases and 10 controls (Table 1). Of these, 22 (73%) were female, 22 (73%) Black, and 30 (100%) non-Hispanic ethnicity. Baseline demographics were similar in cases and controls. However, being immunocompromised was more common in cases, and heart disease was more common in controls. Peak antibody titers were observed in cases at V2 (IgG GMT 642,685), and these declined non-significantly prior to the start of the next RSV season (IgG GMT 396,059, P=0.1306) (Figure 1). Titers then declined significantly 2 years (IgG GMT 134,836, P=0.0003) and 3 years (IgG GMT 117,729, P< 0.0001) after infection. RSV IgG GMTs were not significantly different between cases and controls at any time point. Results were similar when immunocompromised participants were excluded from the analysis.

**Table 1.**
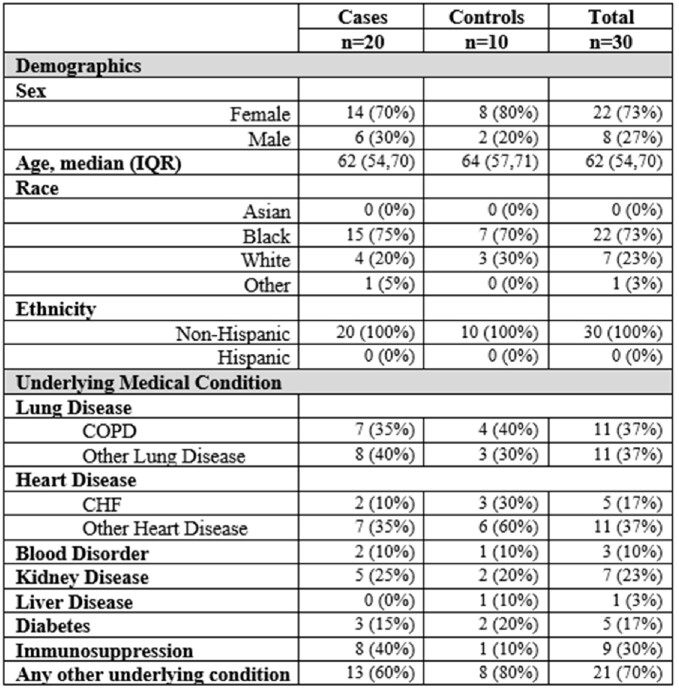
Baseline characteristics of study cohort.

**Figure 1.**
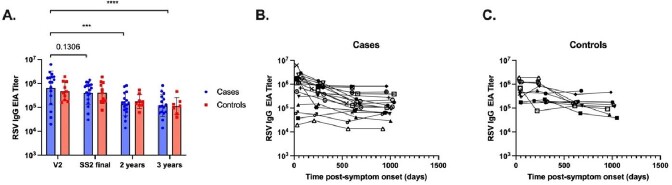
RSV IgG antibody titers by ELISA in (A) Cases vs. Controls; (B) change in titer over time in Cases; and (C) change in titer over time in Controls.

**Conclusion:**

RSV antibody responses peaked in early convalescence and persisted for 3 years after RSV-associated hospitalization. Antibody titers were similarly elevated in cases and controls, indicating the durability of RSV antibodies in older adults.

**Disclosures:**

**Elizabeth Begier, M.D, M.P.H.**, Pfizer: EB is an employee of Pfizer, the sponsor of this study|Pfizer: Stocks/Bonds **Robin Hubler, MS**, Pfizer, Inc.: Employee|Pfizer, Inc.: Stocks/Bonds **Qing Liu, M.S.**, Pfizer Inc.: Stocks/Bonds **Bradford D. Gessner, M.D, M.P.H.**, Pfizer: I am an employee of Pfizer|Pfizer: Stocks/Bonds **Benjamin Lopman, PhD**, Epidemiological Research and Methods, LLC: Advisor/Consultant|Hillevax, Inc: Advisor/Consultant **Nadine Rouphael, MD**, Icon, EMMES, Sanofi, Seqirus, Moderna: Advisor/Consultant **Satoshi Kamidani, MD**, CDC: Grant/Research Support|Emergent BioSolutions: Grant/Research Support|NIH: Grant/Research Support|Pfizer Inc: Grant/Research Support **Evan J. Anderson, MD**, GSK: Advisor/Consultant|GSK: Grant/Research Support|Janssen: Advisor/Consultant|Janssen: Grant/Research Support|Kentucky Bioprocessing, Inc.: Safety Monitoring Board|Moderna: Advisor/Consultant|Moderna: Grant/Research Support|Moderna: Currently an employee|Moderna: Stocks/Bonds|Pfizer: Advisor/Consultant|Pfizer: Grant/Research Support|Sanofi Pasteur: Advisor/Consultant|Sanofi Pasteur: Grant/Research Support|Sanofi Pasteur: Safety Monitoring Board|WCG/ACI Clinical: Data Adjudication Board **Christina A. Rostad, MD**, BioFire Inc.: Grant/Research Support|GlaxoSmithKline Biologicals: Grant/Research Support|Janssen: Grant/Research Support|MedImmune LLC: Grant/Research Support|Meissa Vaccines, Inc.: RSV vaccine technology|Merck & Co., Inc.: Grant/Research Support|Micron Technology, Inc.: Grant/Research Support|Moderna, Inc.: Grant/Research Support|Novavax: Grant/Research Support|PaxVax: Grant/Research Support|Pfizer, Inc.: Grant/Research Support|Regeneron: Grant/Research Support|Sanofi Pasteur: Grant/Research Support

